# Agonist-induced phosphorylation bar code and differential post-activation signaling of the delta opioid receptor revealed by phosphosite-specific antibodies

**DOI:** 10.1038/s41598-020-65589-7

**Published:** 2020-05-22

**Authors:** Anika Mann, Sophia Liebetrau, Marie Klima, Pooja Dasgupta, Dominique Massotte, Stefan Schulz

**Affiliations:** 1 Institute of Pharmacology and Toxicology, Jena University Hospital, Friedrich Schiller University Jena, Drackendorfer Str. 1, 07747 Jena, Germany; 2 Centre de la Recherche Nationale Scientifique, Université de Strasbourg, Institut des Neurosciences Cellulaires et Intégratives, Strasbourg, France

**Keywords:** Phosphorylation, Cellular neuroscience

## Abstract

The δ-opioid receptor (DOP) is an attractive pharmacological target due to its potent analgesic, anxiolytic and anti-depressant activity in chronic pain models. However, some but not all selective DOP agonists also produce severe adverse effects such as seizures. Thus, the development of novel agonists requires a profound understanding of their effects on DOP phosphorylation, post-activation signaling and dephosphorylation. Here we show that agonist-induced DOP phosphorylation at threonine 361 (T361) and serine 363 (S363) proceeds with a temporal hierarchy, with S363 as primary site of phosphorylation. This phosphorylation is mediated by G protein-coupled receptor kinases 2 and 3 (GRK2/3) followed by DOP endocytosis and desensitization. DOP dephosphorylation occurs within minutes and is predominantly mediated by protein phosphatases (PP) 1α and 1β. A comparison of structurally diverse DOP agonists and clinically used opioids demonstrated high correlation between G protein-dependent signaling efficacies and receptor internalization. *In vivo*, DOP agonists induce receptor phosphorylation in a dose-dependent and agonist-selective manner that could be blocked by naltrexone in DOP-eGFP mice. Together, our studies provide novel tools and insights for ligand-activated DOP signaling *in vitro* and *in vivo* and suggest that DOP agonist efficacies may determine receptor post-activation signaling.

## Introduction

The δ-opioid (DOP) receptor, as member of the opioid receptor family, was first discovered in 1975, based on the preference of [Leu]-enkephalin binding to receptors in mouse vas deferens, significantly later followed by the cloning of the single-copy gene *OPRD* for DOP receptor^1–3^. The endogenous enkephalins ([Met]-enkephalin and [Leu]-enkephalin), and the frog skin peptides dermenkephalin and deltorphins I and II were identified as naturally-occurring ligands^4–6^. Deltorphins have high DOP receptor selectivity, whereas enkephalins are moderately DOP receptor-selective^4^. Through coupling to Gα_i_/Gα_0_ proteins, DOP receptor activation leads to inhibition of cAMP production and voltage-gated calcium channels (N- and P/Q-type), as well as induction of β-arrestin signaling and activation of G protein-coupled inwardly rectifying potassium (GIRK) channels^7–10^. In addition, signaling kinases such as ERK, c-Jun N-terminal kinase (JNK), src, Akt, p38 mitogen-activated protein kinase (p38 MAPK) or phospholipase C (PLC) and phospholipase A_2_ (PLA2) are also activated by DOP receptors^11–17^. DOP receptor mRNA and protein are widely expressed throughout the brain, spinal cord and dorsal root ganglia (DRG)^18–21^. The DOP receptor is involved in the regulation of important physiological processes such as thermal and mechanical hyperalgesia, chronic inflammatory pain, anxiety and depression, migraine, locomotion, seizures, emotions, learning and memory, as well as addiction and tolerance development^22–26^. DOP receptor is also involved in wound healing, neuronal, retinal and cardiovascular cytoprotection during hypoxia, as well as cardioprotection during infarct and ischemia^27–29^. Given the more recently discovered DOP receptor expression in peripheral myelinated mechanosensors surrounding hair follicles, DOP receptor may also regulate cutaneous mechanical hypersensitivity^30^.

As a therapeutic target, DOP receptor is under active investigation and appears increasingly attractive because of the global opioid epidemic and its therapeutic potential in pain management, as well as clinical applications in psychiatric and other neurological disorders. Classical opioids like morphine, oxycodone and fentanyl are the most potent clinically used analgesics. However, the prolonged clinical utility of opioids is limited by undesired side effects like constipation, potential for abuse, tolerance development and the potentially fatal risk of respiratory depression^31^. Clinically available opioids exert all their biological effects by interacting with the µ-opioid (MOP) receptor^32^ and all efforts to separate analgesic from undesired pharmacological effects have thus far failed for MOP receptor agonists. This has significantly shifted the research focus to the κ-opioid (KOP) receptor and DOP receptor as potential targets for novel, better-tolerated analgesics. Effective analgesia can be mediated by both receptor subtypes, but stress-induction and dysphoric effects mediated by KOP receptor activation make the DOP receptor a more attractive alternative for the development of new analgesics^33–35^. Besides their inherent analgesic activity, DOP receptor-selective agonists also possess anxiolytic and antidepressant profiles^24,36,37^. Knockout of either DOP receptor or the enkephalin precursor results in anxiety-related responses and depressive-like behaviors in mice^38,39^. Both DOP receptor agonists and antagonist confirmed anxiety-related effects in pharmacological studies. Selective agonists like SNC80 and AR-M1000390 decreased anxiety-related and depressive-like behavior, whereas DOP receptor antagonists produce anxiogenic-like responses in rodents^36,37^. The inhibitory function of DOP receptor agonists on depressive-like behavior is comparable to that of prototypic antidepressant drugs like serotonin reuptake inhibitors or tricyclic antidepressants^36,37,40,41^. This advantageous psychopharmacological profile is desirable in different therapeutic applications and may be important for chronic pain treatment, because of the high comorbidity with anxiety or depression^42^. Besides the positive modulation of emotional tone, DOP receptor agonists are highly effective in inflammatory and neuropathic pain states^23,43,44^ with a reduced side-effect profile in comparison to selective MOP receptor agonists, especially concerning physical dependence, abuse liability, respiratory depression and obstipation. DOP receptor antagonists can also block rewarding properties of morphine, heroin, cocaine, methamphetamine and MDMA^26,45–52^. In contrast to MOP receptor-selective agonists, compounds with high DOP receptor affinity only weakly modulate acute pain^53,54^ but several systemically active DOP receptor agonists were developed as promising alternatives to MOP receptor binding agonists in the treatment of chronic pain^41,55–59^. High DOP receptor expression in DRGs and spinal cord suggested an important function in primary pain processing^18–21^. In peripheral DOP receptor knockout mice, DOP receptor agonists produced increased mechanical sensitivity but showed strongly decreased analgesic effects in chronic inflammatory and neuropathic pain models compared to control mice^60,61^. These findings demonstrated that peripheral DOP receptor signaling is essential to mediate analgesic effects, fostering the hypothesis that peripherally-acting DOP receptor agonists might represent a feasible strategy to treat nociceptive hypersensitivity associated with chronic pain, while avoiding drug-induced central effects. Besides chronic pain research, new DOP receptor-selective compounds were also developed to address other brain disorders. In animal models, DOP receptor agonist were found effective for the treatment of migraine, motor symptoms of Parkinson’s disease, hypoxic/ischemic stress and as a neuroprotective mechanism in Alzheimer’s disease^25,62–65^.

Development and clinical use of new agonists or antagonists targeting DOP receptor requires a profound understanding of DOP receptor regulation at both physiological and molecular levels. Especially, key events of GPCR regulation like receptor phosphorylation, internalization, desensitization and dephosphorylation have been extensively characterized for the closely-related MOP receptor^66–70^ but much less is known about DOP receptor signaling. MOP receptor phosphorylation occurs in an agonist-selective and hierarchical manner at a cluster of four carboxyl-terminal serine (S) and threonine (T) residues, namely T370, S375, T376 and T379. This receptor phosphorylation is mediated by G protein-coupled receptor kinases 2 and 3 (GRK2/3) as well as GRK5^71–73^. Both MOP receptor phosphorylation and internalization were induced in an agonist-specific and time-dependent manner and are determined by a 10 residues sequence in the carboxyl-terminal tail of the receptor. S375, present in the middle of this sequence, is the primary phosphorylation site. Many opioids can stimulate MOP receptor phosphorylation at S375 and on flanking residues (T370, T376, T379), whereas morphine, oxycodone and buprenorphine only induce S375 phosphorylation. Morphine-induced MOP receptor phosphorylation at S375 is mediated by GRK5^71–73^. Multisite-phosphorylation on flanking residues (T370, T376 and T379) requires GRK2/3 and is induced by high-efficacy agonists like DAMGO, fentanyl and etorphine^72,73^. Also, higher-order phosphorylation at T370, T376 and T379 is necessary for opioid-induced MOP receptor internalization^73^.

In the case of DOP receptor, truncation and site-directed mutants suggest that receptor phosphorylation occurs primarily along the C-terminal tail^74–77^. The two S and T residues, T358 and S363, at the end of the C-terminal tail are GRK2 substrates^74,78^. S363 is the primary phosphorylation site induced by highly efficacious agonists like [Met]-enkephalin, DPDPE and SNC80, whereas partial agonists fail to induce DOP receptor phosphorylation^74,75,79–82^. T361 is important for kinase recognition and needed for appropriate T358 and S363 phosphorylation^74^. Also, Glu355 and Asp364 were described as kinase interacting sites, which are necessary for GRK2/GRK3/GRK5 recruitment^83,84^. Studies with receptor mutants demonstrated that DOP receptor phosphorylation at S344 can also undergo heterologous, PKC-mediated phosphorylation^85^. β-arrestin 2 as well as β-arrestin 1 can interact with DOP receptor and induce receptor internalization via clathrin-coated pits in an agonist-dependent manner^86–88^. Following receptor endocytosis, DOP receptor can be either lysosomally downregulated or recycled back to the plasma membrane. Both postendocytotic processes are agonist-dependent^89,90^. Furthermore, receptor dephosphorylation is essential for DOP receptor recycling^91^. So far, DOP receptor dephosphorylation is not well understood and phosphatases involved are still unknown. Furthermore, given the differential functional selectivity among chemically diverse DOP receptor agonists, it is possible that different agonists may induce a hierarchical and agonist-induced phosphorylation pattern after recruitment of one or more GRK isoforms to the receptor. Agonist-induced selective phosphorylation patterns *in vitro* and *in vivo* are poorly understood or unknown.

Here, we have used phosphosite-specific antibodies for T361 and S363 to detect multiple phosphorylated forms of DOP receptor *in vitro* and *in vivo* after treatment with chemically diverse DOP receptor agonists as well as to investigate DOP receptor dephosphorylation in detail.

## Materials and Methods

### Animals

DOP-eGFP mice (8–12 weeks old) were obtained from The Jackson Laboratories (Bar Harbor, Maine, US) and used to detect DOP receptor phosphorylation *in vivo*. Animals were accommodated in small groups, with *ad libitum* access to food and water and under standard laboratory conditions (12 h day/night cycle; lights on at 7:00 a.m.), constant temperature (20–22 °C) and humidity (45–55%). Animals were handled three times before the experiment. Mice were maintained in accordance with the French ministry and the Thuringian state authorities and complied with the *European legislation (directive 2010/63/EU acting on protection of laboratory animals)* and the *European Commission regulations for the care and use of laboratory animals* and were approved by the Landesamt für Verbraucherschutz (number: UKJ-17-039). All methods used were preapproved by the Université de Strasbourg, Institut des Neurosciences Cellulaires et Intégratives and the University Hospital Jena, Institute of Pharmacology and Toxicology (Jena, Germany).

### Plasmids

DNA for human DOP receptor were purchased from cDNA Resource Center (Bloomsberg, PA) and human DOP receptor mutants were generated via gene synthesis and cloned into pcDNA3.1 by Eurofins Genomics (Ebersberg, Germany). The coding sequence for an amino-terminal HA-tag was added.

### Antibodies

The peptide sequence CERVTA-Abu-(pT)PSDG-NH_2_ was used to generate the phosphosite-specific antibody for the T361-phosphorylated form of the DOP receptor. This sequence corresponds to amino acids 355-365 of the human and mouse DOP receptor, respectively. The hemagglutinin peptide sequence YPYDVPDYA was used for the generation of antiserum targeting the HA-tag. The respective peptides were purified by HPLC, coupled to keyhole limpet haemocyanin and conjugates were mixed 1:1 with Freund´s adjuvant and injected into groups of two to four rabbits for anti-pT361 antibody production (5037, 5038) and for anti-HA-tag antibody production (2238, 2239, 2240, 2241). The rabbits were injected at 4-week intervals and serum was obtained 2 weeks after immunizations, beginning with the second injection. Specificity of the antisera was tested using dot blot analysis. For subsequent analysis, antibodies were affinity-purified against their immunizing peptide using a SulfoLink kit (Thermo Scientific, Rockford, IL). In addition, anti-pS363 antibody (A0420) was obtained from Assaybiotech (Fremont, CA, US) and anti-GFP (132002) was purchased from Synaptic Systems (Goettingen, Germany). Anti-GRK2 (sc-562), anti-GRK3 (sc-563), anti-GRK5 (sc-518005) and anti-GRK6 (sc-566) antibodies were obtained from Santa Cruz Biotechnology (Heidelberg, Germany). The anti-HA IgG CF™488 A antibody (SAB4600054) was purchased from Sigma-Aldrich (Steinheim, Germany). Anti-rabbit Alexa488-coupled antibody (A11008) was obtained from Invitrogen (Darmstadt, Germany) and anti-rabbit IgG HRP-coupled (7074) antibody was purchased from Cell Signaling (Massachusetts, US).

### Drugs

SNC80 (ab120684) was obtained from Abcam (Cambridge, UK). DADLE (E7131), fentanyl (F3886), [Met]-enkephalin (M6638), morphin-6-glucuronide (M3528), naloxone (N7758), deltorphin II (T0675), [Leu]-enkephalin (L91333) and phorbol-12-myristat-13-acetat (PMA) (P8139) were purchased from Sigma-Aldrich (Steinheim, Germany). DPDPE (1431), naltrindole (0740), naltribene (0892), naltrexone (0677), AR-M1000390 (4335) and forskolin (1099) were obtained from Tocris (Wiesbaden-Nordenstadt, Germany). Norbuprenorphine (BUP-982-FB) and buprenorphine (BUP-399-HC) were purchased from Lipomed (Arlesheim, Switzerland). ADL5859 (Axon1751) was obtained from Axonmedchem (Groningen, Netherlands). Levomethadone (00424906) was purchased from Sanofi-Aventis (Frankfurt, Germany). Morphine (26-6) was obtained from Merck Pharma (Darmstadt, Germany). Lambda-phosphatase (P0753S) and deltorphin I (sc-396073) were purchased from Santa Cruz (Heidelberg, Germany). Compound 101 (HB2840) was obtained from Hello Bio (Bristol, UK).

### Cell culture and transfection

Human embryonic kidney 293 (HEK293) cells were obtained from DSMZ (Braunschweig, Germany) and AtT20-D16v-F2 (AtT20) cells were purchased from American Type Tissue Culture Collection (Manassas, VA). Both cell types were cultured in Dulbecco’s modified Eagle’s medium (DMEM), supplemented with 10% fetal bovine serum, 2 mM L-glutamine and 100 U/ml penicillin/streptomycin at 37 °C and 5% CO_2_. HEK293 cells and AtT20 cells were stably transfected with HA-tagged DOP receptor or mutant receptor using TurboFect (Thermo Fisher Scientific; Schwerte, Germany). Stable transfected cells were selected in medium supplemented with 400 µg/ml geneticin. To increase the number of stably expressing DOP receptor or receptor mutants in HEK293 cells or AtT20 cells, fluorescence-activated cell sorting was used as described previously^92,93^.

### Small interfering RNA (siRNA) silencing of gene expression

Chemically synthesized double-stranded siRNA duplexes (with 3′-dTdT overhangs) for GRK2 (5′-AAGAAAUUCAUUGAGAGCGAU-3′), GRK3 (5′-AAGCAAGCUGUAGAACACGUA-3′), GRK5 (5′-AAGCAGTATCGAGTGCTAGGA-3′) and GRK6 (5′-AACACCUUCAGGCAAUACCGA-3′) were obtained from Qiagen (Hilden, Germany), PP1α (5′-AAGAGACGCUACAACAUCAAA-3′), PP1β (5′-UACGAGGAUGUCGUCCAGGAA-3′) and PP1γ (5′-AACAUCGACAGCAUUAUCCAA-3′) were purchased from Eurofins Genomics (Ebersberg, Germany) and a non-silencing RNA duplex (5′-GCUUAGGAGCAUUAGUAAA-3′ and 3′-UUUACUAAUGCUCCUAAGC-5′) were obtained from GE Dharmacon (Lafayette, Colorado, US). HEK293 cells stably expressing HA-hDOP were transfected for 3 days with 150 nM siRNA for single transfection or with 100 nM of each siRNA for double transfection using HiPerFect. All experiments showed target protein levels reduced by ≥80%.

### Western blot analysis

Stably HA-hDOP receptor transfected HEK293-cells were plated onto poly-L-lysine-coated 60-mm dishes and grown for 48 h to 80% confluency. Cells were lysed with detergent buffer (50 mM Tris-HCl, pH 7.4; 150 mM NaCl; 5 mM EDTA; 10 mM NaF; 10 mM disodium pyrophosphate; 1% Nonidet P-40; 0.5% sodium deoxycholate; 0.1% SDS) in the presence of protease and phosphatase inhibitors after treatment with agonists, directly. Where indicated, cells were preincubated with antagonists or GRK2/3 inhibitor compound 101 for 30 min before agonist exposure. Cells were centrifuged for 30 min at 4 °C followed by receptor enrichment using wheatgerm-lectin-agarose beads. Samples were inverted for 2 h at 4 °C. Where indicated, DOP receptor was dephosphorylated using lambda protein phosphatase (Santa Cruz; Heidelberg, Germany) for 1 h at 30 °C. After washing three times, proteins were eluted using SDS sample buffer for 30 min at 50 °C. Proteins were separated on 7.5% or 12% SDS-polyacrylamide gels. After electroblotting, membranes were incubated with either 0.1 µg/ml anti-pT361 (5038) or anti-pS363 antibodies over night at 4 °C. Subsequently, blots were washed followed by detection using enhanced chemiluminescence detection (ECL) (Thermo Fisher Scientific; Schwerte, Germany) of bound antibodies. Blots were thereafter stripped and reprobed with the anti-HA antibody (2238) to ensure equal loading of the gels.

### *In vivo* phosphorylation studies

First, the ability of different DOP receptor agonists to induce receptor phosphorylation *in vivo* was investigated. DOP-eGFP mice (n = 3 per compound) were given injections of DOP receptor agonists SNC80 (10 mg/kg, i.p.), ADL5859 (100 mg/kg, p.o.), AR-M1000390 (60 mg/kg, p.o.), DOP receptor antagonist naltrexone (10 mg/kg, i.p.), or vehicle. 15 minutes after treatment mice were sacrificed. Immediately brains were dissected out, frozen on dry ice and stored at -80 °C until biochemical analysis. Second, a dose-response study for SNC80 to induce DOP receptor phosphorylation was performed. Increasing doses of SNC80 (0.3, 1, 3, 10 mg/kg) (n = 3 per dose) were injected i.p. for 15 min. Brains were dissected out and transferred into ice-cold detergent buffer (50 mM Tris-HCL, pH 7.4; 150 mM NaCl; 5 mM EDTA; 10 mM NaF; 10 mM disodium pyrophosphate; 1% Nonidet P-40; 0.5% sodium deoxycholate; 0.1% SDS; containing protease and phosphatase inhibitors). After sample homogenization using a MINILYS workplace homogenizer (Peqlab; Erlangen, Germany), brains were lysed for 1 h at 4 °C followed by centrifugation at 16000 × g for 30 min at 4 °C. Supernatants were immunoprecipitated with anti-GFP beads (NanoTag; Goettingen, Germany) for 2 h at 4 °C. Protein concentration was determined using a Bradford Assay Kit (Thermo Fisher Scientific, Schwerte, Germany) and proteins were eluted from the beads with SDS-sample buffer for 30 min at 50 °C. Subsequently, proteins were separated on 7.5% SDS-polyacrylamide gels and after electroblotting membranes were incubated with anti-pT361 (5038) or anti-pS363 antibodies at a concentration of 0.1 µg/ml. After ECL detection of bound antibodies, blots were stripped and reprobed with the anti-GFP antibody (Synaptic Systems; Goettingen, Germany) to confirm equal loading of the gels.

### Analysis of DOP receptor internalization

HEK293-cells stably expressing HA-tagged DOP or receptor mutants were plated onto poly-L-lysine-coated coverslips and grown for 24 h. Next, cells were incubated with rabbit anti-HA antibody (2238) in serum-free medium for 2 h at 4 °C. Cells were fixed with 4% paraformaldehyde and 0.2% picric acid in phosphate buffer (pH 6.9) for 30 min at room temperature after agonist or antagonist exposure for 30 min at 37 °C. Subsequently, cells were washed three times with phosphate buffer (22.6 ml/L 1 M NaH_2_PO_4_•H_2_O; 77.4 ml/L 1 M Na_2_HPO_4_•H_2_O; 0.1% Triton X-100, pH 7.4) and permeabilized. After incubation with an Alexa488-coupled goat anti-rabbit antibody (Invitrogen; Darmstadt, Germany), cells were mounted and receptor internalization was examined using a Zeiss LSM510 META laser scanning confocal microscope (Jena, Germany). For quantitative internalization assays, stably HA-hDOP receptor transfected HEK293-cells were plated onto 24-well plates and grown overnight. After preincubation with anti-HA antibody (2238) for 2 h at 4 °C, cells were exposed to agonists or antagonists for 30 min at 37 °C and subsequently fixed for 30 min at room temperature. Cells were washed several times with PBS and incubated with a peroxidase-conjugated secondary antibody (Santa Cruz; Heidelberg, Germany). After additional washing steps, the HRP-substrate ABTS was added and optical density was measured at 405 nm using an iMark™ Microplate Absorbance Reader (BioRad, Munich, Germany).

### Membrane potential assay

Membrane potential change was measured as previously described^94^. Stably HA-hDOP receptor expressing AtT20 cells were plated into 96-well plates. After 48 h, cells were washed with Hank´s balanced salt solution (HBSS), buffered with 20 mM HEPES pH 7.4, containing 1.3 mM CaCl_2_; 5.4 mM KCl; 0.4 mM K_2_HPO_4_; 0.5 mM MgCl_2_; 0.4 mM MgSO_4_; 136.9 mM NaCl; 0.3 mM Na_2_HPO_4_; 4.2 mM NaHCO_3_; 5.5 mM glucose. Afterwards, cells were incubated with membrane potential dye (FLIPR Membrane Potential kit BLUE, Molecular Devices, Biberach, Germany) for 45 min at 37 °C. The final injection volume of compounds or vehicle was 20 µl and the initial volume in the wells was 180 µl (90 µl buffer and 90 µl dye). Finally, 20 µl of compound was added to the cells (final volume in the well was 200 µl resulting in a 1:10 dilution of the compound). The compounds were prepared at 10x concentrations. Compounds or buffer were injected after 60 seconds baseline measurement. Right after injection of compounds or vehicle membrane potential change was measured at 37 °C using a FlexStation 3 microplate reader (Molecular Devices; Biberach, Germany). The buffer-only trace for each corresponding data point was subtracted from the data after normalization to the baseline.

### Data analysis

Protein bands detected on Western blots were quantified using ImageJ 1.47 v software (National Institute of Health, Bethesda, MD, USA). Data (Western blots and ELISA) were analyzed using GraphPad Prism 5 software (La Jolla, CA, USA). Statistical analysis was carried out with *one-way ANOVA* followed by Bonferroni correction. *P* values < 0.05 were considered statistically significant. Dose response curves were analyzed and compiled with OriginPro.

## Results

### Characterization of phosphosite-specific antibodies to analyze agonist-induced phosphorylation of T361 and S363 in the carboxyl-terminal tail of DOP receptor

**Figure 1:**
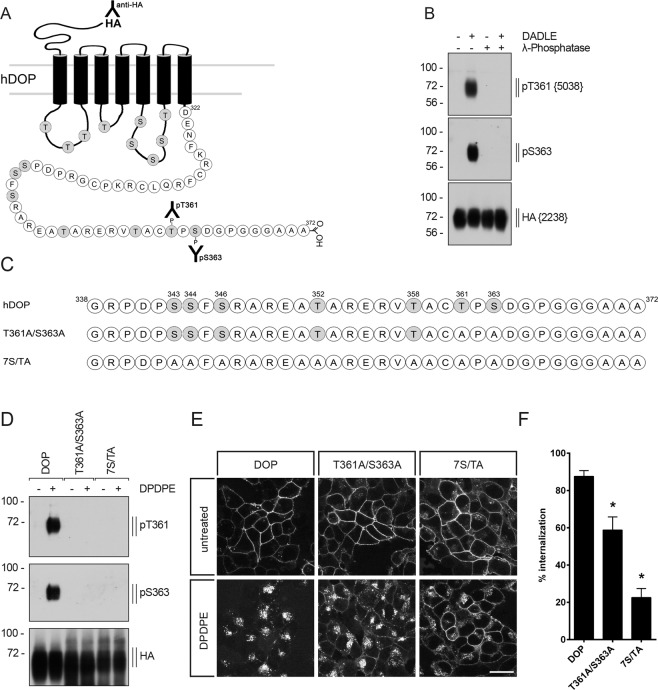
Characterization of phosphosite-specific DOP receptor antibodies using λ-phosphatase and receptor mutants. (**A**) Schematic representation of the human DOP receptor (hDOP). Potential intracellular phosphorylation sites are depicted in gray. T361 was targeted for the generation of the phosphosite-specific anti-pT361 antibody and anti-pS363 antibody was acquired commercially. **(B)** Characterization of phosphosite-specific antibodies directed against T361 and S363 using λ-phosphatase. Stably HA-tagged hDOP-receptor expressing HEK293 cells were either not treated (−) or treated (+) with 10 µM DADLE for 10 min. Lysates were then either not incubated (−) or incubated (+) with λ-phosphatase and immunoblotted with the phosphosite-specific antibodies anti-pT361 {5038} or anti-pS363. Blots were stripped and reprobed with the anti-HA antibody {2238} as a loading control. Blots are representative, n = 3. Molecular mass markers (kDA) are indicated, left. **(C)** Sequence of the carboxyl-terminal tail of hDOP receptor showing all potential phosphorylation sites. Serine (S) and threonine (T) residues indicated were exchanged to alanine. **(D)** HEK293 cells stably expressing HA-hDOP receptor, T361A/S363A or 7 S/TA were not treated (−) or treated (+) with 1 µM DPDPE for 10 min and lysates were immunoblotted with the antibodies to pT361 or pS363. Blots were stripped and reprobed with the anti-HA antibody. Blots are representative, n = 5. **(E)** Cells described in (D) were preincubated with antibody to HA-tag and subsequently exposed to 10 µM DPDPE or vehicle for 30 min at 37 °C. Cells were fixed, permeabilized, immunofluorescently stained and examined using confocal microscopy. Images are representative from one of three independent experiments. Scale bar, 20 µm. **(F)** Receptor internalization was quantified by ELISA. Cells described in (D) were preincubated with antibody to HA-tag and stimulated with 10 µM DPDPE or vehicle at 37 °C for 30 min. Cells were fixed and labeled with a peroxidase-conjugated secondary antibody. Receptor internalization was measured by enzyme-linked immunosorbent assay and quantified as the percentage of internalized receptors in DPDPE-treated cells. Data are mean ± SEM of five independent experiments performed in quadruplicate. Results were analyzed by one-way ANOVA followed by Bonferroni’s post-hoc test (*p < 0.05).

There are several potential phosphate acceptor sites in the intracellular loops and within the carboxyl-terminal tail of human DOP receptor (Fig. 1A). To examine the temporal and spatial dynamics of DOP receptor phosphorylation in its carboxyl-terminal tail, we generated a polyclonal phosphosite-specific antibody for the carboxyl-terminal residue T361, and we used the commercially available anti-S363 antibody (Fig. 1A). In addition, we generated an anti-HA antibody which binds to an amino-terminally fused hemagglutinin epitope tag (HA-tag) in order to detect HA-DOP receptor independent of phosphorylation status (Fig. 1A). First, antisera were affinity purified against their immunizing peptides and specificity was then verified with corresponding synthetic peptides using dot-blot assays. All antibodies, which clearly detected their respective peptide were further characterized using Western blot analysis. The anti-pT361 [5038] and anti-pS363 antibodies specifically detected the respective T361- or S363-phosphorylated form of DOP receptor after DADLE stimulation of HEK293 cells stably transfected with human HA-DOP receptor (Fig. 1B). Both phosphosite-specific antibodies were no longer able to detect their cognate forms of phosphorylated DOP receptor after treatment with lambda phosphatase (Fig. 1B), but the receptor protein was still detectable using the anti-HA antibody [2248] (Fig. 1B). Different DOP receptor mutants were generated for further characterization of the phosphosite-specific antibodies (Fig. 1C). As expected, no phosphorylation signal for pT361 and pS363 was detectable after DPDPE stimulation in the T361A/S363A mutant as well as after global mutation of all serine and threonine residues present in the carboxyl-terminal tail (7S/T-A) (Fig. 1D). Also, internalization after DPDPE incubation was reduced in the T361A/S363A mutant (Fig. 1E,F). A stronger inhibition of DOP receptor internalization was detectable in the 7S/TA mutant (Fig. 1E,F). These results show that the phosphosite-specific antibodies directed against T361 and S363 in the carboxyl-terminal tail clearly recognize only the respective phosphorylated form of DOP receptor and that the mutation of only T361 and S363 is sufficient to diminish DOP receptor internalization.

### DOP receptor phosphorylation and internalization occur in a time- and concentration-dependent manner with S363 as primary phosphorylation site

**Figure 2:**
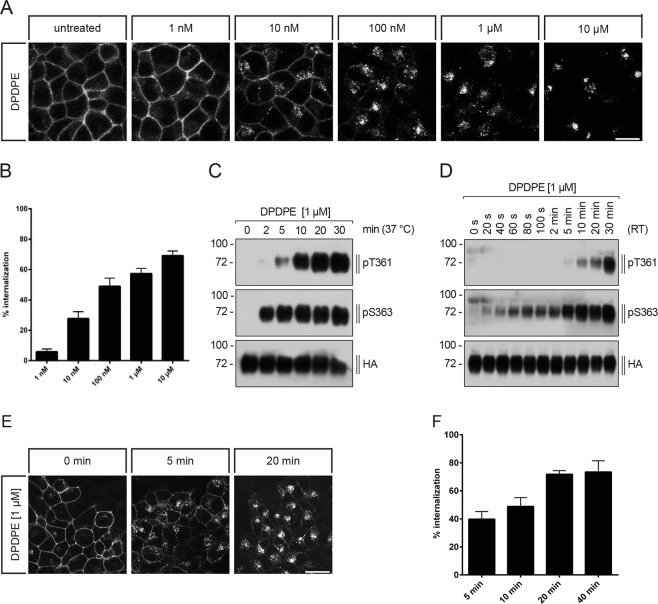
Time course of DPDPE-induced DOP receptor phosphorylation, concentration-dependent DOP receptor phosphorylation and internalization. (**A**) HEK293 cells stably expressing HA-tagged hDOP receptor were preincubated with anti-HA antibody and stimulated with the indicated DPDPE concentrations for 30 min at 37 °C. After stimulation, cells were fixed, permeabilized, immunofluorescently stained and examined using confocal microscopy. Images are representative from one of three independent experiments. Scale bar, 20 µm. **(B)** Cells described in (A) were preincubated with anti-HA antibody and stimulated with the indicated DPDPE concentrations for 30 min at 37 °C. After fixation, cells were labeled with a peroxidase-conjugated secondary antibody and receptor internalization was measured by enzyme-linked immunosorbent assay. Receptor internalization was measured by ELISA and quantified as the percentage of internalized receptors compared to untreated cells. Data are means ± SEM of four independent experiments performed in quadruplicate. **(C,D)** Cells described in (**A**) were exposed to 1 µM DPDPE for the indicated times and temperatures (**C**) at 37 °C, (**D**) at 22 °C (room temperature, RT) and lysates were immunoblotted with anti-pT361 or anti-pS363 antibodies. Blots are representative of n = 4 (**C**) or n = 5 (**D**) independent experiments. **(E)** Cells described in (A) were preincubated with anti-HA antibody and thereafter stimulated with 1 µM DPDPE for the indicated durations at 37 °C. Cells were fixed, permeabilized, immunofluorescently stained and examined using confocal microscopy. Images are representative from one of three independent experiments. Scale bar, 20 µm. **(F)** Cells described in (**A**) were preincubated with anti-HA antibody and stimulated with 10 µM DPDPE for the indicated durations at 37 °C. Cells were fixed and labeled with peroxidase-conjugated secondary antibody. Receptor internalization was measured by ELISA and quantified as the percentage of internalized receptors in agonist-treated cells compared to untreated cells. Data are mean ± SEM of five independent experiments performed in quadruplicate.

We then examined the DPDPE-induced DOP receptor internalization using fluorescence microscopy and for quantification we used a cell-surface enzyme-linked immunosorbent assay (ELISA). DOP receptor internalization was initiated after treatment with 10 nM DPDPE and reached a maximum after incubation with 10 µM DPDPE (Fig. 2A,B). We then examined the time-course of DPDPE-induced T361 and S363 phosphorylation and receptor internalization. After DPDPE stimulation, a robust phosphorylation at S363 was detectable within 2 min, which remained at high levels throughout the 30 min treatment period. T361 phosphorylation was first detectable after 5 min following DPDPE incubation and increased throughout the 30 min treatment period (Fig. 2C). To determine the DOP receptor phosphorylation time-course in more detail, agonist was added to the cells at room temperature (RT) for shorter time periods. Under these conditions, S363 phosphorylation occurred within 40 s, whereas T361 phosphorylation became first detectable after 10 min, suggesting that S363 is the primary site of phosphorylation, followed by T361 (Fig. 2D).

Internalization of DOP receptor was first detectable after 5 min DPDPE treatment and reached a maximum after 20 min (Fig. 2E,F). DPDPE-induced DOP receptor phosphorylation and internalization occur in a time-dependent manner with S363 as primary phosphorylation site followed by T361. Also, receptor internalization takes place in a concentration-dependent manner.

### DOP receptor phosphorylation is mediated by GRK2 and GRK3

**Figure 3:**
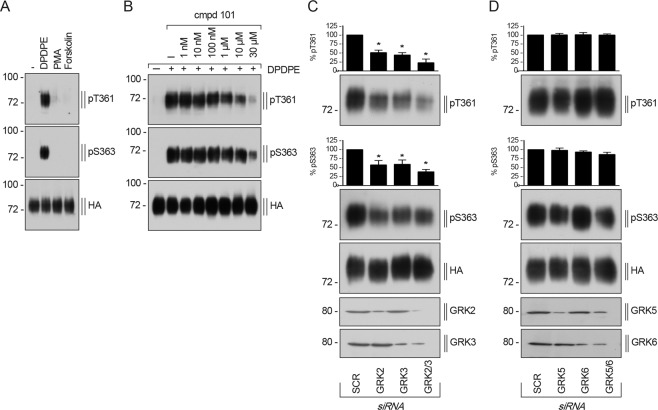
DPDPE-induced DOP receptor phosphorylation is mediated by GRK2 and GRK3. (**A**) HEK293 cells stably expressing HA-hDOP receptor were stimulated with 1 µM DPDPE, 1 µM PMA or 10 µM forskolin for 10 min at 37 °C. Cell lysates were immunoblotted with anti-pT361 or anti-pS363 antibodies. Blots were stripped and reprobed with the anti-HA antibody as loading control. Blots are representative, n = 4. **(B)** Cells described in (**A**) were preincubated with either vehicle (DMSO; -) or compound (cmpd) 101 at the indicated concentrations for 30 min at 37 °C, then treated with buffer or 1 µM DPDPE for 10 min at 37 °C. Lysates were immunoblotted as described in (**A**). Blots are representative, n = 3. **(C,D)** Cells described in (**A**) were transfected with siRNA targeted either to **(C)** GRK2, GRK3, or GRK2 and GRK3 (GRK2/3), **(D)** GRK5, GRK6, or GRK5 and GRK6 (GRK5/GRK6), or non-silencing siRNA control (SCR) for 72 h, then stimulated with1 µM DPDPE for 10 min at 37 °C. Lysates were immunoblotted as described in (A). Knock down of GRKs was confirmed by Western blot (bottom panels in C and D). Densitometry readings, above the blots, were normalized to SCR (control) transfected cells, which were set to 100%. Data are means ± SEM from three to five independent experiments. *p < 0.05 vs. SCR control by one-way ANOVA with Bonferroni’s post-hoc test.

Phosphorylation of GPCRs can be mediated by different types of kinases, G protein-coupled receptor kinases (GRKs) and second messenger-activated kinases (e.g. PKA, PKC). To examine if DOP receptor phosphorylation could also be mediated heterologously by PKA or PKC, we therefore incubated cells with phorbol-12-myristat-13-acetat (PMA) or forskolin. Neither forskolin nor PMA induced any detectable phosphorylation at T361 or S363 (Fig. 3A). To evaluate which GRK isoforms are mediating the DPDPE-induced DOP receptor phosphorylation, we used the chemical GRK2/3 selective inhibitor compound 101 (cmpd101) as well as siRNA knockdown experiments. DPDPE-induced phosphorylation at T361 and S363 is reduced in a concentration-dependent manner after inhibition of GRK2/3 activation using compound 101 (Fig. 3B). Treatment with specific GRK2 or GRK3 siRNA sequences also led to a significant reduction of DPDPE-induced phosphorylation at T361 and S363 (Fig. 3C). It is possible that the loss of either GRK2 or GRK3 could be compensated for by the remaining isoform, because of the close relationship between the two GRK isoforms. Therefore, we evaluated the inhibitory effect of siRNA knockdown of both GRK2 and GRK3 on DOP receptor phosphorylation. A combination of siRNA knockdown of both GRK isoforms produced a stronger inhibition of T361 and S363 phosphorylation, indicating that GRK2 and GRK3 function as a redundant system for DPDPE-induced DOP receptor phosphorylation (Fig. 3C). To rule out that also GRK5 and GRK6 were involved in DPDPE-induced DOP receptor phosphorylation, we performed the same siRNA knockdown experiments for the two GRK isoforms. As expected, knockdown of GRK5, GRK6 or a combination of both, could not reduce the DPDPE-induced phosphorylation signal neither at T361 nor at S363 (Fig. 3D). These results suggest that GRK2 and GRK3 are responsible for DPDPE-induced DOP receptor phosphorylation at T361 and S363.

### DOP receptor agonists induce varying levels of receptor phosphorylation and internalization

**Figure 4:**
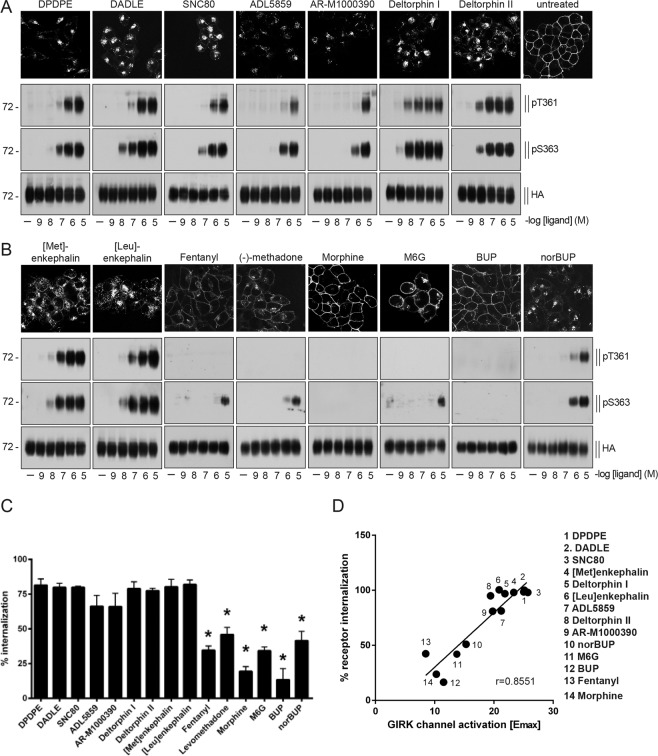
Agonist-induced DOP receptor phosphorylation and internalization. (**A,B** top) Stably HA-tagged hDOP receptor-expressing HEK293 cells were preincubated with anti-HA antibody, followed by stimulation with 10 µM DPDPE, DADLE, SNC80, ADL5859, AR-M1000390, deltorphin I, deltorphin II, [Met]-enkephalin, [Leu]-enkephalin, fentanyl, (−)-methadone, morphine, morphine-6-glucuronide (M6G), buprenorphine (BUP), norbuprenorphine (norBUP) or vehicle for 30 min at 37 °C. Cells were fixed, permeabilized, immunofluorescently stained, and subsequently examined using confocal microscopy. Images are representative, n = 3. Scale bar, 20 µm. **(A,B** bottom) HEK293 cells stably expressing HA-hDOP receptor were stimulated with the compounds listed in (A) or vehicle at concentrations ranging from 10^-9^ to 10^-5^ M for 10 min at 37 °C. Lysates were immunoblotted with antibodies to pT361 or pS363. Blots were stripped and reprobed with the anti-HA antibody. Blots are representative, n = 3-5. **(C)** Cells described in (**A**) were preincubated with anti-HA antibody and stimulated with vehicle or 10 µM of compounds listed in (**A**) for 30 min at 37 °C. Cells were fixed and labeled with a peroxidase-conjugated secondary antibody. Receptor internalization was measured by ELISA and quantified as the percentage of internalized receptors in agonist-treated cells compared to untreated cells. Data are means ± SEM of five independent experiments performed in quadruplicate. Results were analyzed by one-way ANOVA followed by Bonferroni’s post-hoc test (*p < 0.05). **(D)** Correlation between G protein-mediated (GIRK channel activation, E_max_) and arrestin-mediated (internalization, % of untreated control cells) agonist effects in HA-hDOP receptor-transfected HEK293 cells (internalization) or AtT-20 cells (GIRK channel activation). Solid line, linear regression analysis revealed a correlation coefficient r = 0.8551.

We next surveyed a large selection of chemically diverse selective DOP receptor ligands and clinically used opioids for their ability to induce DOP receptor phosphorylation and internalization. We consistently observed that endocytotic activity of these selective DOP receptor agonists and common opioids correlated with their ability to induce receptor phosphorylation at T361 and S363 (Fig. 4). Robust phosphorylation and receptor internalization were detectable after treatment with DPDPE, DADLE, SNC80, ADL5859, AR-M1000390, deltorphin I, deltorphin II, [Met]-enkephalin, [Leu]-enkephalin and norbuprenorphine (Fig. 4). Fentanyl and (−)-methadone as well as morphine-6-glucuronide, an active metabolite of morphine, induced phosphorylation only at S363, whereas morphine and buprenorphine failed to induced DOP receptor phosphorylation (Fig. 4). The DOP-selective agonists ADL5859 and AR-M1000390 had been previously described as non- or weakly internalizing agonists^79,95^. A significantly higher concentration (10 µM) was used for all compounds in order to distinguish between DOP agonists which induces a strong, partial or no internalization. These results confirm the consensus model that GPCR phosphorylation is highly correlated with, and a prerequisite for, subsequent internalization. Correlation analysis between G protein-dependent (GIRK activation) and arrestin-dependent (internalization) effects of the tested compounds revealed positive linear correlation (r = 0.8551; Fig. 4D), suggesting that DOP receptor agonists do not display any bias in intracellular coupling.

### Chemically diverse DOP receptor agonists show varying efficacies in GIRK channel activation (G protein-dependent signaling)

**Figure 5:**
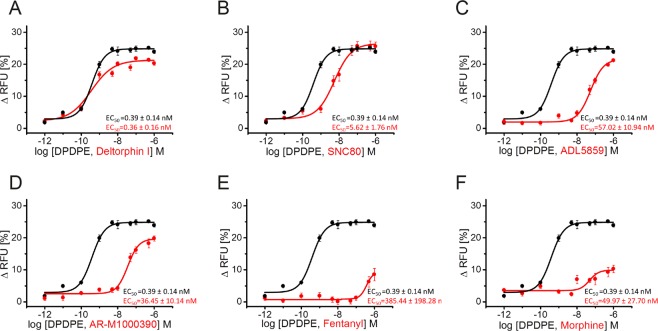
G protein signaling of diverse DOP receptor agonists. AtT-20 cells stably expressing HA-hDOP receptors were stimulated with vehicle, or DPDPE, deltorphin I **(A)**, SNC80 **(B)**, ADL5859 **(C)**, AR-M1000390 **(D)**, fentanyl **(E)** or morphine **(F)** at a concentration range of 10^−6^ to 10^−13^ M and GIRK channel activation was measured in a membrane potential assay. Dose response curves were calculated with OriginPro using sigmoidal non-linear fitting for data from three independent experiments performed in duplicate (mean ± SEM). Vehicle-induced changes in fluorescence signal (background) were subtracted from signals obtained using agonist-containing solutions. The DPDPE graph is the same in every panel and serve as a comparator. RFU, relative fluorescence units.

A previously described fluorescence-based membrane potential assay, that detects Gβγ-mediated activation of inwardly rectifying potassium (GIRK) channels was used to examine G protein signaling of DOP receptor at high temporal resolution^94^. We studied the ability of different DOP receptor ligands to activate GIRK channels (Fig. 5, Table 1). Notably, deltorphin II (EC_50_ of 0.11 ± 0.09 nM) and DADLE (EC_50_ of 0.16 ± 0.04 nM) were the most potent agonists tested and compared to DPDPE (EC_50_ of 0.39 ± 0.14 nM) (Table 1). Deltorphin I exhibited a similar dose-response curve with EC_50_ values of 0.36 ± 0.16 nM (Fig. 5A, Table 1). [Met]-enkephalin and [Leu]-enkephalin are similarly potent agonists with EC_50_ values of 0.78 ± 0.08 nM and 0.77 ± 0.15 nM (Table 1), as well as SNC80, buprenorphine and norbuprenorphine with EC_50_ values of 5.62 ± 1.76 nM, 3.36 ± 2.13 nM and 10.67 ± 1.46 nM, respectively (Fig. 5B, Table 1). ADL5859 and AR-M1000390 exhibited partial agonist activity with EC_50_ values of 57.02 ± 10.94 nM and 36.45 ± 10.14 nM (Fig. 5C,D). Morphine and fentanyl showed only a weak activity with a remarkably reduced maximal effect compared to DPDPE (Fig. 5E,F; Table 1). Together with our results from phosphorylation and internalization studies, these observations suggest that DOP agonists range from partial to full agonism without intracellular signaling bias (Fig. 4D).

**Table 1:** G protein signaling of diverse DOP receptor agonists.

	EC_50_ (nM)	E_max_	E_max_ in % of DPDPE
DPDPE	0.39 ± 0.14	25.14 ± 0.35	100%
DADLE	0.16 ± 0.04	23.34 ± 1.70	92.8%
Deltorphin I	.36 ± 0.16	21.86 ± 0.50	87.0%
Deltorphin II	0.11 ± 0.09	19.43 ± 0.53	77.3%
[Met]-enkephalin	0.78 ± 0.08	25.03 ± 0.78	99.6%
[Leu]-enkephalin	0.77 ± 0.15	20.88 ± 0.70	83.1%
SNC-80	5.62 ± 1.76	25.72 ± 1.41	102.3%
ADL5859	57.02 ± 10.94	21.23 ± 0.39	84.4%
AR-M1000390	36.45 ± 10.14	19.77 ± 0.78	78.6%
Buprenorphine	n.d.	11.5 ± 1.6	42.3%
Norbuprenorphine	43.8 ± 20.7	15.3 ± 1.50	56.3%
Morphine	49.97 ± 27.70	10.27 ± 1.16	40.9%
Fentanyl	385.44 ± 198.28	8.49 ± 1.92	33.8%
M6G	1200.05 ± 400.70	13.73 ± 0.86	54.6%

n.d. not detectable.

### DPDPE-induced phosphorylation and internalization is inhibited by the DOP receptor antagonist

**Figure 6:**
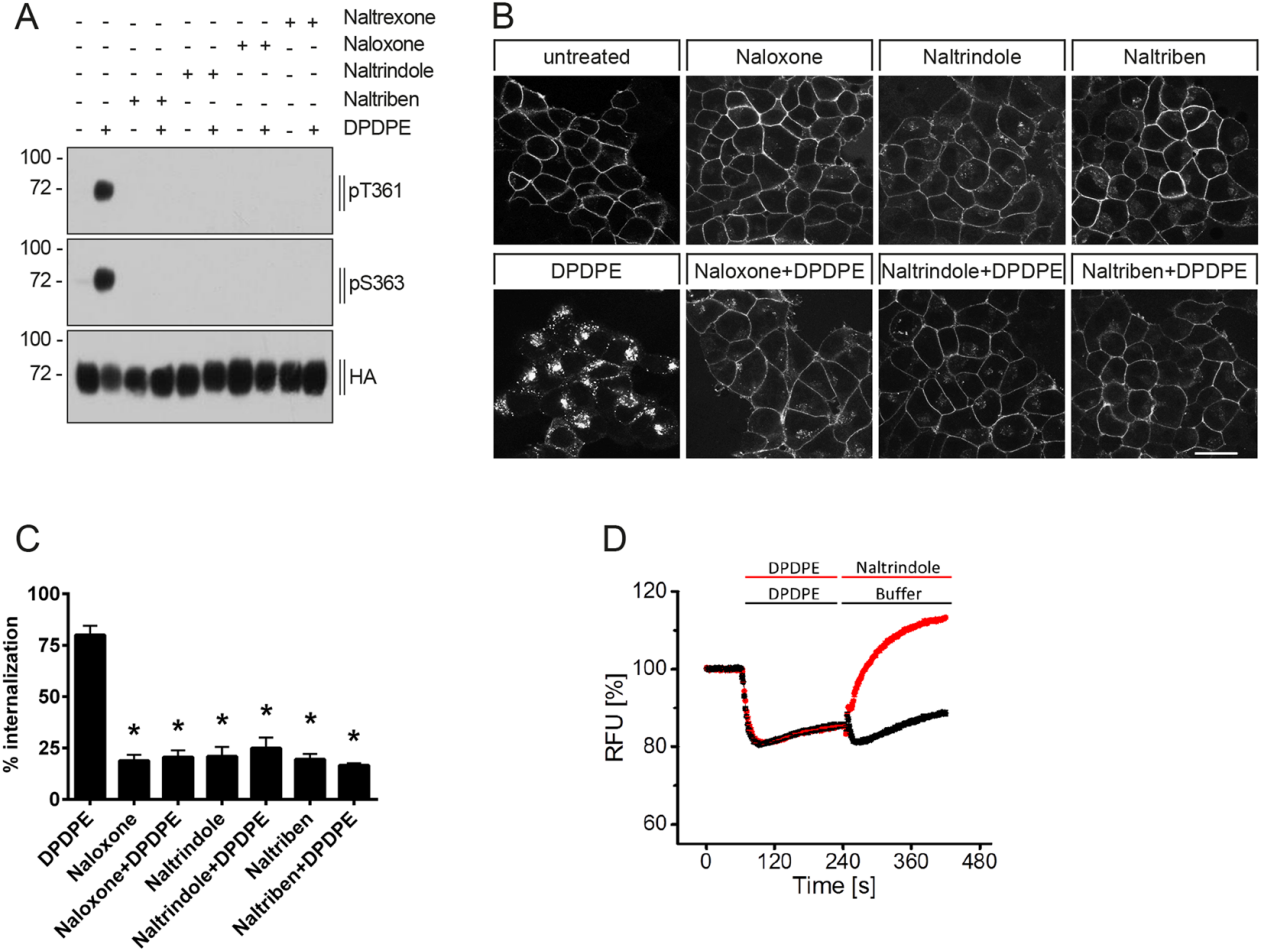
Antagonist-selective inhibition of DPDPE-induced DOP receptor phosphorylation, internalization and G protein signaling. (**A**) Stably HA-hDOP receptor-expressing HEK293 cells were either not preincubated (−) or preincubated (+) with 5 µM naloxone, naltrindole, naltriben or naltrexone for 30 min at 37 °C, then stimulated with vehicle (water, -) or with 1 µM DPDPE (+) for 10 min at 37 °C. Cell lysates were then immunoblotted with antibodies to pT361 or pS363. Blots were stripped and reprobed with the anti-HA antibody. Blots are representative, n = 4. **(B)** Cells described in (**A**) were preincubated with anti-HA antibody and then treated with vehicle (DMSO), 5 µM naloxone, naltrindole or naltriben and with or without 1 µM DPDPE for 10 min at 37 °C. After fixation, cells were permeabilized, immunofluorescently stained and examined using confocal microscopy. Images are representative, n = 3. Scale bar, 20 µm. **(C)** Cells described in (**A**) were preincubated with anti-HA antibody and stimulated with vehicle (DMSO), 5 µM naloxone, naltrindole or naltriben and with or without 1 µM DPDPE for 10 min at 37 °C. Cells were then fixed and labeled with a peroxidase-conjugated secondary antibody. Receptor internalization was measured by ELISA and quantified as percentage of internalized receptors in agonist-treated cells. Data are means ± SEM from five independent experiments performed in quadruplicate. *p < 0.05 vs. DPDPE by one-way ANOVA with Bonferroni post-hoc test. (**D**) Reversal of DPDPE-induced hyperpolarization by naltrindole using a fluorescence-based membrane potential assay. After baseline recording for 60 sec, HA-hDOP receptor -expressing AtT-20 cells were exposed to 1 µM DPDPE and 240 sec later, 10 µM naltrindol was added, yielding a final molar DPDPE/antagonist ratio of 1:10. Shown are the normalized traces obtained from the average of four individual experiments performed in triplicates.

Both, the non-selective opioid receptor antagonist naloxone and naltrexone and the selective DOP receptor antagonists naltrindole^96–98^ and naltriben^99–101^ block DPDPE-induced phosphorylation at T361 and S363 as well as receptor internalization (Fig. 6A–C). Moreover, addition of naltrindole induced a reversal of the DPDPE-induced hyperpolarization towards baseline level in the GIRK channel activation assay (Fig. 6D).

### Agonist-selective DOP receptor phosphorylation is also observed in vivo in mouse brain

**Figure 7:**
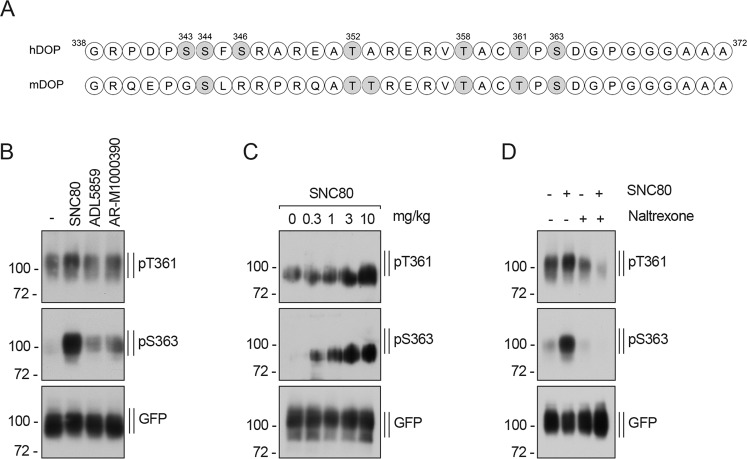
Agonist-induced DOP receptor phosphorylation in mouse brain. (**A**) Schematic representation of the human (h) and mouse (m) DOP receptor C-terminal tail. All potential phosphate acceptor sites are depicted in gray. **(B)** After injection of 0.9% NaCl (i.p.), 10 mg/kg SNC80 (i.p.), 100 mg/kg ADL5859 (p.o.) or 60 mg/kg AR-M1000390 (p.o.) for 15 min, DOP-eGFP knock-in mice. were euthanized and brains were removed. DOP receptor was immunoprecipitated with anti-GFP protein agarose beads and immunoblotted with antibodies to pT361 or pS363. Blots were stripped and reprobed for GFP. Blots are representative, n = 3. **(C)** As in (**B**), but mice were treated with SNC80 (i.p.) at the indicated doses for 15 min. Blots are representative, n = 3. **(D)** As in (B), but mice were treated with 10 mg/kg naltrexone or SNC80 alone or pretreated with naltrexone for 15 min followed by SNC80 (10 mg/kg, i.p.). Blots are representative, n = 3. Positions of molecular mass markers are indicated on the left (in kDa).

The fact that corresponding mouse DOP receptor phosphorylation sites are located at equivalent positions compared to the human DOP receptor (Fig. 7A) enabled us to use the phosphosite-specific antibodies to analyze agonist-induced phosphorylation *in vivo* in more detail. DOP-eGFP mice were treated with different types of systemically active DOP receptor ligands. The selective DOP full agonist SNC80 induced a strong and concentration-dependent phosphorylation at T361 and S363 which could be blocked by naltrexone (Fig. 7B–D), similar to observations made *in vitro* for this combination of compounds (Suppl. Fig. 1). A weaker increase of *in vivo* DOP phosphorylation signal was detectable after administration of ADL5859 and AR-M1000390 in comparison to saline treatment (Fig. 7B). However, the ADL5859 and AR-M1000390 doses used were much higher than behaviorally necessary (effective dose in most behavior assays: 10 mg/kg AR-M1000390 and ADL5859^95,102,103^) (Fig. 7B), albeit inducing weaker receptor phosphorylation than SNC80. Therefore, we selected SNC80 for dose-response *in vivo* experiments (Fig. 7C). These results indicate that DOP receptor phosphorylation occurs after stimulation by selective agonists in a concentration-dependent manner *in vivo*. We also observed significant constitutive phosphorylation of T361 (unstimulated controls in Fig. 7B–D) that could be related to the previously reported high constitutive activity of DOP receptors^104^.

### DOP receptor dephosphorylation occurs in a time-dependent manner with S363 as primary dephosphorylation site

**Figure 8:**
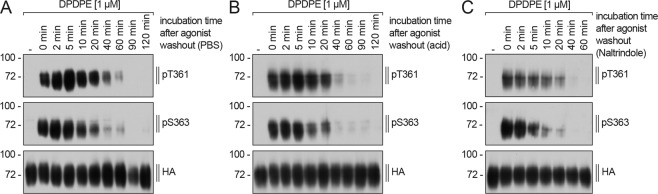
Time-course of DOP receptor dephosphorylation. (**A–C**) Stably HA-hDOP receptor expressing HEK293 cells were treated with 1 µM DPDPE for 10 min at 37 °C, three times washed with(**A**) PBS, (**B**) citrate buffer or (**C**) citrate buffer containing naltrindole and then incubated in the absence of DPDPE in serum-free medium for the indicated times at 37 °C. Lysates were immunoblotted with antibody to pT361 or pS363. Blots were stripped and reprobed with the anti-HA antibody. Blots are representative, n = 3.

Phosphorylation at S363, the primary phosphorylation site, occurred during the first minute whereas a phosphorylation signal at T361 was first detectable after prolonged stimulation with DPDPE (10 min). Therefore, we examined DOP receptor dephosphorylation, using three different buffer, in order to investigate if distinct temporal dynamics exist between both phosphorylation sites. Washout with citric acid buffer removes high-affnity agonsits and disrupt receptor-ligand-binding more efficiently than phosphate buffer^105^. Naltrindole was added to citric acid buffer to further facilitate displacement of DPDPE from the receptor and thus terminate agonist stimulation. Cells were washed with citric acid after DPDPE stimulation and then incubated in agonist-free medium with or without naltrindole to differentiate the dephosphorylation time in more detail. Dephosphorylation of T361 and S363 occurred more quickly after washout with citric acid buffer and naltrindole. Interestingly, no differences were observed in dephosphorylation time between T361 and S363 (Fig. 8). These results indicate that there is no primary site of dephosphorylation.

### Dephosphorylation of DOP receptor is mediated by PP1

**Figure 9:**
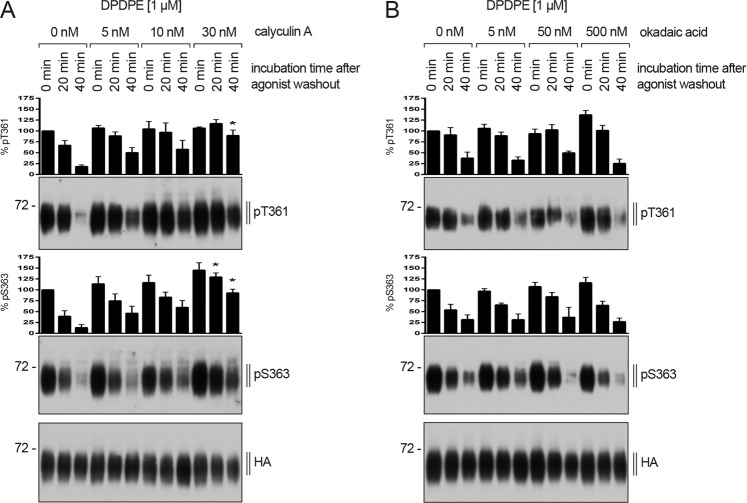
Inhibition of DOP receptor dephosphorylation by calyculin A but not okadaic acid. (**A,B**) HEK293 cells stably expressing HA-hDOP receptor were either preincubated with the indicated concentrations of (**A**) calyculin A or (**B**) okadaic acid for 30 min at 37 °C and then stimulated with 1 µM DPDPE for 10 min at 37 °C. Subsequently, cells were washed three times with ice cold citrate buffer and then incubated in serum-free medium in the absence of DPDPE for 0, 20 or 40 min in the presence of the above indicated concentrations of **(A)** calyculin A or **(B)** okadaic acid at 37 °C. Lysates were immunoblotted with antibody to pT361 or pS363. Blots were stripped and reprobed with the anti-HA antibody. Densitometry readings, above the blots, were normalized to those in DPDPE-stimulated cells (0 nM and 0 min), which were set to 100%. Data are mean ± SEM from three independent experiments. *p < 0.05 vs. DPDPE controls by one-way ANOVA with Bonferroni’s post-hoc test.

Classified by their catalytic subunits, seven families of serine/threonine-specific protein phosphatases, PP1-PP7, have been identified^106–108^. Calyculin A is an inhibitor of PP1 and PP2 activity to a similar extent^108–110^. In contrast, okadaic acid can block the activity of PP2, PP4, and PP5, but has little effect on PP1 activity^108–114^. Both inhibitors are not able to reduce the activity of PP3. When cells stably expressing DOP receptor were exposed to escalating concentrations of calyculin A or okadaic acid, DOP receptor dephosphorylation was inhibited in a concentration-dependent manner only by calyculin A (Fig. 9A,B). Thus, the present results strongly suggest that PP1 activity is required for DOP receptor dephosphorylation at T361 and S363.

**Figure 10:**
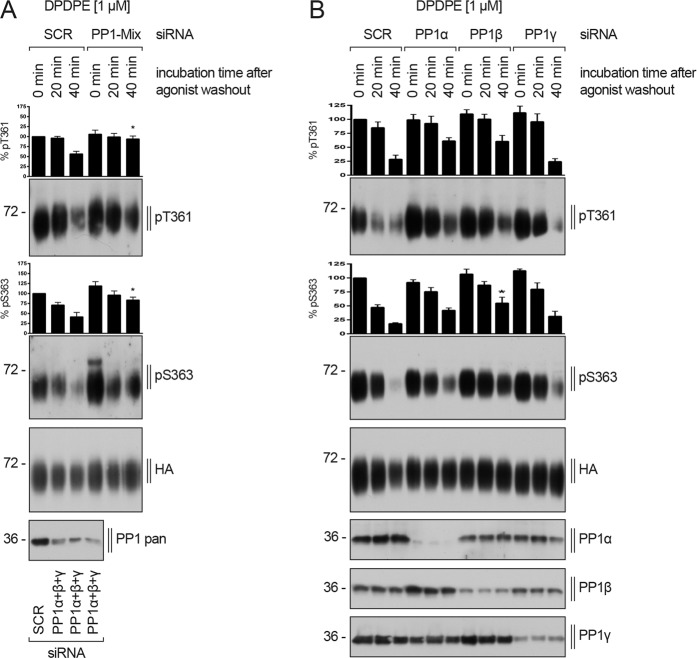
DOP receptor dephosphorylation is mediated by PP1. (**A–C**) HEK293 cells stably expressing HA-hDOP receptor were transfected with siRNA targeting either **(A)** a combination of PP1α, PP1β and PP1γ (PP1-Mix), or **(B)** PP1α, PP1β, or PP1γ, or non-silencing siRNA control (SCR) for 72 h. After stimulation with 1 µM DPDPE for 10 min at 37 °C, cells were washed three times with cold citrate buffer and then incubated in serum-free medium in the absence of DPDPE for indicated times at 37 °C. Lysates were immunoblotted with antibody to pT361 or pS363. Blots were stripped and reprobed with the anti-HA antibody. Knock down of protein expression by siRNA was confirmed by Western blot blot (bottom panels in **A,B**). Densitometry readings, above the blots, were normalized to those in SCR-transfected cells, which were set to 100%. Data are mean ± SEM from three to four independent experiments. *p < 0.05 vs. SCR by one-way ANOVA with Bonferroni’s post-hoc test.

We next performed siRNA knockdown experiments to confirm these results and to evaluate the contribution of the catalytic subunits PP1α, PP1β and PP1γ to DOP receptor dephosphorylation. Simultaneous knockdown of all three PP1 catalytical subunits nearly completely blocked DOP receptor dephosphorylation in DPDPE-treated cells (Fig. 10A). Only inhibition of PP1α and PP1β expression resulted in a robust reduction of dephosphorylation at T361 and S363 (Fig. 10B). In contrast, PP1γ siRNA knockdown did not attenuate T361 and S363 dephosphorylation (Fig. 10B). Moreover, inhibition of PP2α and PP2β had no effect on DOP receptor dephosphorylation (data not shown). These results confirmed that PP1 activity was required for efficient DOP receptor dephosphorylation, most likely mediated by PP1α and/or PP1β.

## Discussion

In the present study, we used phosphosite-specific antibodies for DOP to analyze distinct phosphorylation patterns induced by a large variety of selective DOP agonist and opioids, both *in vitro* and *in vivo*. Moreover, we detected hierarchical and temporally controlled receptor multisite phosphorylation and dephosphorylation. Appropriate signaling by GPCRs is dependent on the specific activation of canonical regulatory kinases and phosphatases that together create the overall signaling output. Agonist binding to the receptor triggers activation and signaling through its associated heterotrimeric G protein which involves GRKs or second messenger-dependent protein kinases, such as PKC or PKA. GRK-imprinted phosphorylation barcodes increase the affinity for β-arrestin binding, which uncouple the receptor from its G protein, regulate receptor internalization and subsequent desensitization while simultaneously initiating β-arrestin-dependent signaling. Internalized GPCRs are either sorted to lysosomes for degradation or recycle back to the plasma membrane for reinsertion. Return of GPCRs to their resting state requires dissociation of agonist and β-arrestins but also dephosphorylation of the receptor.

Agonist-induced phosphorylation usually involves a specific pattern of serine and threonine residues and is considered the first step in receptor regulation. Receptor phosphorylation was studied in great detail for several GPCRs, most notably the β2-adrenoceptor (β2-AR), MOP and NOP receptor as well as somatostatin sst2, sst3 and sst5 receptor subtypes^71–73,92,93,115–124^. A primary phosphate-acceptor site has been identified for each GPCR, and phosphorylation often correlates with receptor internalization levels. The prevailing hypothesis that complete GPCR phosphorylation is required for maximal internalization is supported by these observations and a hallmark of full agonists. In case of DOP receptor, mutagenesis studies have localized multiple serine and threonine residues located in the carboxyl-terminal domain as major phosphorylation sites^74–76^. Using phosphosite-specific antibodies, we found that DOP receptor phosphorylation proceeds in a temporal hierarchy, with S363 as primary phosphorylation site followed by T361 phosphorylation. Stimulation with DPDPE induced maximal receptor internalization with a similar kinetic as seen for MOP receptor^72,121^. Mutation of T358, T361 and S363 has no influence on the capacity to recruit β-arrestins^84^ but reduced receptor internalization which was abolished when all serine and threonine residues in the C-terminal tail were mutated to alanine. Earlier studies have shown, that T358 and S363 are substrates for GRK2^74,78^. The data presented here are in line with these previous findings and also demonstrate that T361 is phosphorylated by GRK2/3 after DPDPE stimulation *in vitro*. In addition, DOP receptor can also undergo heterologous site-specific PKC-dependent phosphorylation at S344^85^. Accordingly, we did not observe any impact on T361 or S363 phosphorylation level either after PKC- or PKA-activation.

Like MOP receptor agonists^71–73^, most DOP receptor agonists stimulated receptor phosphorylation to a degree that correlated with the level of internalization. The enkephalin analogs DPDPE and DADLE are highly selective DOP receptor agonists^125,126^. Both compounds induced robust activation of GIRK channels, phosphorylation and receptor internalization equally to the natural DOP receptor agonists, the enkephalins and deltorphins. SNC80 is a full agonist in G protein-dependent cAMP assays with high DOP receptor selectivity and antinociceptive, antidepressant and anxiolytic properties, but also producing convulsions^36,37,40,55,127–130^. We found that SNC80 also induced robust GIRK channel activation and showed strong DOP receptor phosphorylation. Our data confirmed previous studies which also showed DOP internalization after SNC80 exposure^79,128,130^. The SNC80 derivative AR-M1000390 and the spirocyclic agonist ADL5859 were previously described as potent, highly selective and orally available DOP receptor agonists^41,131^. Both compounds reduced inflammatory and neuropathic pain and were devoid of proconvulsive activity^79,95,131,132^. We found that ADL5859 and AR-M1000390 induced GIRK channel activation with similar potency and produced significant DOP receptor phosphorylation and internalization at saturating concentrations *in vitro*. However, in DOP-eGFP knock-in mice both compounds were not able to induce DOP receptor internalization *in vivo*^79,95,102^. It should be mentioned that we used unphysiologically high concentrations of both compounds to see whether they can elicit any DOP receptor phosphorylation or internalization. Therefore, our *in vitro* observations are not comparable to the *in vivo* situation. However, it was at least demonstrated for ADL5859 and AR-M1000390 that substantial DOP receptor internalization can occur at very high concentrations *in vitro*^79^. In contrast, fentanyl and (−)-methadone failed to induce any robust activation of GIRK channels but produced phosphorylation at DOP receptors only at S363, but not at T361, followed by weak receptor internalization. Morphine failed to induce any phosphorylation at DOP receptor and induced only a weak activation in G protein-mediated GIRK assays. Previous studies had shown that DOP receptor was not internalized after morphine exposure, which is consistent with the present findings^81,133^. Glucuronate conjugation at the 6-position in morphine is known to enhance DOP receptor binding^134^. Conversely, we found DOP receptor phosphorylation after morphine-6-glucuronide exposure followed by weak receptor internalization. Buprenorphine has a high binding affinity to DOP receptor^135^ but no receptor phosphorylation and internalization was detectable in our hands, whereas norbuprenorphine, the major active metabolite of buprenorphine, induced DOP receptor phosphorylation and receptor internalization. Together our data show a strong correlation between GIRK channel activation, phosphorylation and DOP receptor internalization (Fig. 4D). These correlation is also supported by a previous study which showed, that DOP receptor stimulation, GIRK channel undergo arrestin-dependent internalization^136^.

Localization of DOP receptor has been studied using electron microscopy, immunohistochemical detection and in situ hybridization^19–21,137–141^. Here, fine-tuning of *in vivo* phosphorylation of DOP receptor in brain was analyzed for the first time using Western blot. The same techniques were used in recent studies to analyze phosphorylation at MOP and NOP receptors^72,93,120,121^. Both residues, T361 and S363, are phosphorylated *in vivo* in a dose-dependent manner after agonist injection. Interestingly, T361 is also constitutively phosphorylated which may reflect high constitutive activity of DOP receptors that had been reported before^104^. In comparison to animals injected with saline, *in vivo* phosphorylation in mouse brains was only weakly increased by ADL5859 and AR-M1000390, which may explain the lack of internalization as well as the absence of proconvulsive activity observed previously^129^.

So far, the molecular mechanisms of DOP receptor dephosphorylation have never been investigated. Here we used phosphosite-specific antibodies in combination with siRNA knock-down screening to identify phosphatases involved in DOP receptor dephosphorylation. We identified PP1α and PP1β as phosphatases which catalyzed T361 and S363 dephosphorylation after agonist removal. Inhibition of PP1α or PP1β expression resulted in increased DPDPE-driven receptor phosphorylation at both residues. Dephosphorylation of T361 occurs slower than dephosphorylation at S363. In comparison, dephosphorylation of DOP receptor occurs at a much slower rate than that observed for MOP receptor and sst5^72,124^. Dephosphorylation of MOP receptor and sst5 involves PP1γ, dephosphorylation of sst2 requires PP1β activity and dephosphorylation of sst3 involves PP1α and PP1β^72,92,124,142^. Our findings indicate that PP1γ mediated dephosphorylation is linked to GPCRs that recycle rapidly to the plasma membrane. PP1 consists of one catalytic subunit and one or more regulatory subunits. The substrate specificity and subcellular localization of PP1s is determined by the regulatory subunit, of which more than 40 exist^106^. However, it remains unclear which mechanisms regulate phosphatase specificity at GPCRs. It is conceivable that carboxyl-terminal phosphorylation sites, specific sequences in the intracellular loops or β-arrestin trafficking patterns may all contribute to selection of phosphatases.

In conclusion, we identified for the first time a specific and hierarchical agonist-induced phosphorylation pattern in the carboxyl-terminal domain of DOP receptor *in vitro* and *in vivo*. The phosphorylation pattern correlates with receptor internalization and conceivably provides evidence for a general biochemical mechanism by which the different functional effects of DOP receptor agonists are achieved. Further, differential agonist-induced multi-site phosphorylation patterns suggest that chemically diverse agonists induce distinct receptor conformations that could explain differences in DOP receptor agonist efficacy. This study provides important tools to characterize agonist-dependent regulation of DOP receptor signaling at the cellular and intact animal leyel, which will facilitate the development of DOP receptor agonists for selected therapeutic indications.

## Supplementary information

Supplementary Dataset 1.

## Data Availability

The datasets in the current study are available from the corresponding author on reasonable request.
