# Supplementary material for: Agonist-induced phosphorylation bar code and differential post-activation signaling of the delta opioid receptor revealed by phosphosite-specific antibodies
Source: Sci Rep. 2020 May 22;10:8585. doi: 10.1038/s41598-020-65589-7 (PMC7244497; doi:10.1038/s41598-020-65589-7)

**Agonist-induced phosphorylation bar code and differential post-activation signaling of the delta opioid receptor revealed by phosphosite-specific antibodies**

Anika Mann^1*^, Sophia Liebetrau^1^, Marie Klima^1^, Pooja Dasgupta^1^, Dominique Massotte^2^, Stefan Schulz^1*^

**Supp 1: Naltrexone inhibition of SNC80-induced DOP receptor phosphorylation *in vitro*.** Stably HA-hDOP receptor-expressing HEK293 cells were either not preincubated (-) or preincubated (+) with 5 µM naltrexone for 30 min at 37 °C, then stimulated with vehicle (water, -) or with 1 µM SNC80 (+) for 10 min at 37 °C. Cell lysates were then immunoblotted with antibodies to pT361 or pS363. Blots were stripped and reprobed with the anti-HA antibody. Blots are representative, n=4.


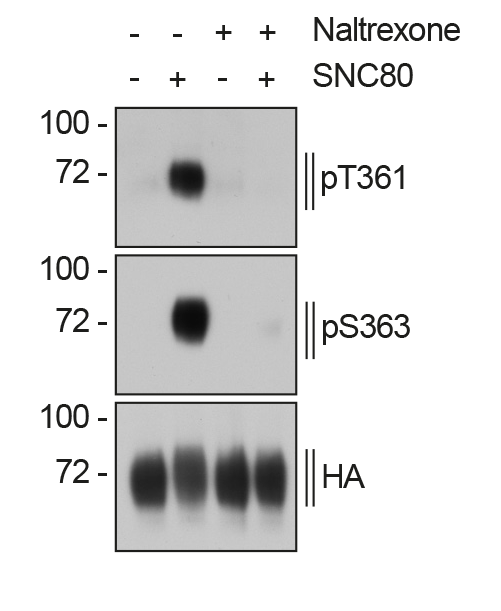

Supplement: Supplementary file 1 — Supplementary Dataset 1. [file 41598_2020_65589_MOESM1_ESM.docx]
